# Analytical and Clinical Validation of Expressed Variants and Fusions From the Whole Transcriptome of Thyroid FNA Samples

**DOI:** 10.3389/fendo.2019.00612

**Published:** 2019-09-11

**Authors:** Trevor E. Angell, Lori J. Wirth, Maria E. Cabanillas, Maisie L. Shindo, Edmund S. Cibas, Joshua E. Babiarz, Yangyang Hao, Su Yeon Kim, P. Sean Walsh, Jing Huang, Richard T. Kloos, Giulia C. Kennedy, Steven G. Waguespack

**Affiliations:** ^1^Division of Endocrinology, Diabetes and Metabolism, Keck School of Medicine, University of Southern California, Los Angeles, CA, United States; ^2^Department of Medicine, Massachusetts General Hospital, Boston, MA, United States; ^3^Department of Endocrine Neoplasia and Hormonal Disorders, The University of Texas MD Anderson Cancer Center, Houston, TX, United States; ^4^Otolaryngology–Head & Neck Surgery, Oregon Health & Science University, Portland, OR, United States; ^5^Department of Pathology, Brigham and Women's Hospital and Harvard Medical School, Boston, MA, United States; ^6^Research and Development, Veracyte, South San Francisco, CA, United States; ^7^Medical Affairs, Veracyte, South San Francisco, CA, United States; ^8^Research and Development, Medical Affairs, and Clinical Affairs, Veracyte, South San Francisco, CA, United States

**Keywords:** molecular diagnostics, thyroid cancer, fine-needle aspiration, thyroid molecular assays, RNA-sequencing, transcriptome, atypia of undetermined significance, follicular neoplasm

## Abstract

**Introduction:** The Afirma® Xpression Atlas (XA) detects gene variants and fusions in thyroid nodule FNA samples from a curated panel of 511 genes using whole-transcriptome RNA-sequencing. Its intended use is among cytologically indeterminate nodules that are Afirma GSC suspicious, Bethesda V/VI nodules, or known thyroid metastases. Here we report its analytical and clinical validation.

**Methods:** DNA and RNA were purified from the same sample across 943 blinded FNAs and compared by multiple methodologies, including whole-transcriptome RNA-seq, targeted RNA-seq, and targeted DNA-seq. An additional 695 blinded FNAs were used to define performance for fusions between whole-transcriptome RNA-seq and targeted RNA-seq. We quantified the reproducibility of the whole-transcriptome RNA-seq assay across laboratories and reagent lots. Finally, variants and fusions were compared to histopathology results.

**Results:** Of variants detected in DNA at 5 or 20% variant allele frequency, 74 and 88% were also detected by XA, respectively. XA variant detection was 89% when compared to an alternative RNA-based detection method. Low levels of expression of the DNA allele carrying the variant, compared with the wild-type allele, was found in some variants not detected by XA. 82% of gene fusions detected in a targeted RNA fusion assay were detected by XA. Conversely, nearly all variants or fusions detected by XA were confirmed by an alternative method. Analytical validation studies demonstrated high intra-plate reproducibility (89%-94%), inter-plate reproducibility (86–91%), and inter-lab accuracy (90%). Multiple variants and fusions previously described across the spectrum of thyroid cancers were identified by XA, including some with approved or investigational targeted therapies. Among 190 Bethesda III/IV nodules, the sensitivity of XA as a standalone test was 49%.

**Conclusion:** When the Afirma Genomic Sequencing Classifier (GSC) is used first among Bethesda III/IV nodules as a rule-out test, XA supplements genomic insight among those that are GSC suspicious. Our data clinically and analytically validate XA for use among GSC suspicious, or Bethesda V/VI nodules. Genomic information provided by XA may inform clinical decision-making with precision medicine insights across a broad range of FNA sample types encountered in the care of patients with thyroid nodules and thyroid cancer.

## Introduction

Genomic assessment for precision medicine is a story of incredible advancement. In the Nineteenth century, observations of dividing cancer cells suggested that they were abnormal clones caused by defects of hereditary material (1). The first isolation of a specific DNA variant responsible for cancer formation was in 1982, a G>T substitution in codon 12 of the *HRAS* gene (1). Since then there has been an explosion of cancer genome understanding, with the documentation of more than 350 cancer driving genes and 100,000 somatic mutations by the early Twenty-first century (1). Subsequently, a common theme that has emerged in oncology suggests that each cancer can be genomically subtyped and that the downstream gene expression profile predicts its cellular morphology, clinical presentation, prognosis, and that this presents an opportunity for the development of effective targeted therapies.

Investigation and understanding of benign and malignant thyroid nodules has followed a similar course as many insights have been gained from large genomic studies across a spectrum of histologic subtypes (2–14). Concomitantly, targeted therapies for advanced thyroid cancer have emerged, including FDA approved or investigational selective inhibitors of *AKT*^1^, ALK (15, 16), *BRAF*^1^ (16, 17), *cKIT*^1^, *EGFR*^2^, *HRAS*^3^,^4^,^5^,^6^ (18), *KRAS*^2, 4, 5, 6^,^7^,^8^,, *MET*^1^, *mTOR* (16, 19), *NRAS*^1, 4, 5, 6^, *NTRK* (15, 16, 20), *PAX8/PPARG*^9^, *PIK3CA* (PI3K) (21), *PTEN*^1^, *RET* (16, 22, 23), *ROS1* (15, 16), and microsatellite instability-high or mismatch repair deficient solid tumors (24).

Currently, an important tool to avoid unnecessary diagnostic surgery among cytologically indeterminate thyroid nodules is the Afirma Genomic Sequencing Classifier (GSC) (25, 26). In this context, “cytologically indeterminate” refers to the two Bethesda categories Atypia of Undetermined Significance/ Follicular Lesion of Undetermined Significance (“Bethesda III”) and Follicular Neoplasm/ Suspicious for a Follicular Neoplasm (Bethesda IV) (27) or their equivalents (28). The Afirma GSC is a cancer rule-out test that partners whole transcriptome RNA sequencing genomic information derived from a fine-needle aspiration (FNA) biopsy with machine learning to create algorithms that identify specific neoplasms, including MTC (see Table 1 for histology subtype abbreviations), and ultimately classify the sample as GSC benign or suspicious. Nodules identified as GSC benign have a cancer risk of approximately 4% and can be considered for clinical observation *in lieu* of diagnostic surgery (29–31). Conversely, GSC suspicious nodules have an increased cancer risk of approximately 50%, which is roughly 2-fold higher than it was based on cytology alone. These nodules are typically considered for surgical resection (29–31). The GSC algorithms rely heavily on differential gene expression for sample classification. Several of the included modules make limited use of RNA-sequencing's ability to detect genomic variants and fusions in the transcribed RNA, including *BRAF* V600E variants, and *RET/PTC1* and *RET/PTC3* fusions, which are all highly predictive of malignancy. However, it was not until the introduction of the Afirma Xpression Atlas (XA) that variant and fusion identification by RNA-sequencing was more significantly harnessed. The use of whole transcriptome sequencing by both the Afirma GSC and Afirma XA allows the same sample collection and shipping method to be used for both tests, and both tests are run on the same FNA sample. Using one sample for both tests facilitates successful test results despite the small genomic sample obtained by FNA. XA findings may predict tissue cellular morphology, clinical syndromes, cancer behavior (including mode of metastasis), prognosis, and facilitate the selection of effective targeted therapy in the appropriate clinical setting.

**Table 1:** Histopathology subtypes.

**Malignant subtypes**	
FC	Follicular carcinoma. Variants include capsular invasion (FC-c) and vascular invasion (FC-v)
FVPTC	Follicular variant of papillary thyroid carcinoma. Variants include FVPTC micro carcinomas (mFVPTC)
HCC	Hürthle cell carcinoma. Variants include capsular invasion (HCC-c) and vascular invasion (HCC-v)
PTC	Papillary thyroid carcinoma. Variants include PTC micro carcinomas (mPTC), tall-cell variant (PTC-TCV), and tall-cell variant micro carcinomas (mPTC-TCV)
MTC	Medullary thyroid carcinoma
PDC	Poorly differentiated carcinoma
WDC-NOS	Well-differentiated carcinoma not otherwise specified
**Benign subtypes**	
BFN	Benign follicular nodule
CLT	Chronic lymphocytic thyroiditis (aka, Hashimoto's thyroiditis). Also known as LCT (lymphocytic thyroiditis)
FA	Follicular adenoma
HCA	Hürthle cell adenoma
HN	Hyperplastic nodule
HTA	Hyalinizing trabecular adenoma
FT-UMP	Follicular tumor with unknown malignant potential
WDT-UMP	Well-differentiated tumor with unknown malignant potential

Here we describe the analytical and clinical validation of XA to report nucleotide variants and gene fusions beyond *BRAF* V600E, *RET/PTC1*, and *RET/PTC3* fusions using whole transcriptome RNA-seq data derived from FNA samples. We compared the XA results to a targeted DNA panel for nucleotide variants and compared XA results to a targeted RNA fusion panel for gene/gene fusion detection. The data demonstrate a high level of agreement between methods and that these variant and fusion methods alone cannot serve as rule-out test to exclude cancer/noninvasive follicular thyroid neoplasm with papillary-like nuclear features (NIFTP). When used among Bethesda III/IV nodules that are GSC suspicious, Bethesda V/VI thyroid nodules, or by extension known thyroid cancer metastases (data not shown), the genomic content provided by XA can provide additional information that may inform clinical decision-making.

## Materials and Methods

### Nucleic Acid Extraction

RNA and DNA were extracted from thyroid FNAs or control tissues (described below under Cohorts and Controls, respectively) using the Qiagen AllPrep Micro Kit (Qiagen, Hilden, Germany) according to manufacturer's instructions. RNA was quantitated using Quantiflour (Promega, Madison, WI) and DNA was quantitated using PicoGreen (Promega, Madison, WI). Fluorescence was read on a Tecan Infinite M200 Pro (Tecan, Männedorf, Switzerland). RNA Integrity Number (RIN) was determined for RNA using RNA Pico Chips on the Bioanalyzer 2100 (Agilent, Santa Clara, CA).

### Custom AmpliSeq Panels

The Ion AmpliSeq Designer (Thermo Fisher, Waltham, MA) was used to generate a custom DNA panel against 568 targets described in Pagan et al. (6). Nine Y chromosome SNPs were included to assign genomic gender, which was compared to clinical gender to ensure sample identity. A custom RNA AmpliSeq Variant Panel was designed against the same targets described for the DNA AmpliSeq panel. A custom AmpliSeq RNA Fusion Panel was also generated (Thermo Fisher, Waltham, MA), with 168 fusions plus 6 house-keeping genes for controls.

### AmpliSeq Library Preparation

Ion Torrent Libraries were generated using the Ion AmpliSeq Library Kit 2.0 (Thermo Fisher, Waltham, MA) according to manufacturer's instructions using 10 ng input material. RNA was first reverse transcribed with SuperScript IV VILO (Thermo Fisher, Waltham, MA). Following cleanup, libraries were quantitated using qPCR on a QuantStudio 6 (Thermo Fisher, Waltham, MA), normalized to 100 pM, and loaded onto the IonChef (Thermo Fisher, Waltham, MA). Pooled libraries were loaded onto the Ion 540 Chip (Thermo Fisher, Waltham, MA) and sequenced on the Ion Torrent S5XL (Thermo Fisher, Waltham, MA).

### Controls

Each DNA plate included the Horizon Quantitative Multiplex Reference Standard (HD701, Horizon, Cambridge, United Kingdom), NA12878 (Coriell, Camden, NJ), *BRAF* V600E positive thyroid tissue (Cooperative Human Tissue Network, National Cancer Institute, Bethesda, MD), and *TERT* C228T positive thyroid tissue (Asterand, Westbury, NY).

### Targeted DNA/RNA Sequencing Data Analysis

The Ion Torrent Suite Software version 5.6.0 (Thermo Fisher, Waltham, MA) was used to demultiplex, map reads to the reference genome hg19, and call variants. Specifically, tmap version 5.6.8 was used for reads mapping, and Torrent Variant Caller version 5.6–10 was used to detect variants with parameter settings optimized for low frequency variant detection with minimal false negative calls on Ion AmpliSeq experiments.

### Targeted RNA Sequencing for Fusion Data Analysis

The Ion Reporter version 5.6.0 was used to detect fusions. Key fusion detection parameters were set as follows: minimum 20,000 total valid mapped reads to qualify a sample for further analysis; minimum 20 reads required to call a fusion; medium sensitivity, which requires 70% overlap between reads and reference sequence with at-least 66.66% exact matches in the overlap.

### qPCR Data

Taqman assays were obtained from Thermo Fisher or designed and synthesized by Integrated DNA Technologies (IDT, Coralville, IA). Ten nanogram of RNA was reverse transcribed with QuantiTect (Qiagen, Hilden, Germany). qPCR reactions were performed in duplicate using Fast Advanced Master Mix (Thermo Fisher, Waltham, MA), VIC-labeled primer-limited TBP (Hs00427620_m1, Thermo Fisher), and the FAM-labeled fusion-specific taqman assay (Thermo Fisher or IDT). qPCR assays were run on the QuantStudio 6 (Thermo Fisher, Waltham, MA) and Ct values were determined with the QuantStudio Real Time Software v1.3.

### RNA-Seq Data

RNA-seq data was generated using the TruSeq RNA Exome kit (formerly RNA Access, Illumina, San Diego, CA) using 15 ng of total RNA as previously described (25). Libraries were sequenced on the NextSeq 500 platform (Illumina, San Diego, CA).

### RNA-Seq Data Analysis

Fastq files were aligned to hg19 using STAR aligner, version 2.4.1b (32). Fusions were called using STAR-fusion version 0.5.4 (33). Variants were called using GATK version 3.3 (34), following the best practices for variant calling on RNA-seq (35, 36).

### Fusion Nomenclature

All fusion partners are described in their 5′/3′ order. In the commercial Afirma XA report, all fusion partners are reported alphabetically except CCDC6/RET and NCOA4/RET, which are reported using their colloquial names of RET/PTC1 and RET/PTC3, respectively.

### Cohorts

943 blinded FNA samples with sufficient DNA were utilized from the following sources: Afirma GSC Algorithm Training set (*n* = 32, 217, 75, 35, 36 from Bethesda categories II-VI, respectively) (25), the prospectively collected, and multicenter Afirma GSC clinical validation cohort (*n* = 152 Bethesda III/IV, *n* = 17 Bethesda II and *n* = 29 Bethesda V/VI) (25), samples with paired castPCR *BRAF* V600E truth Bethesda V (*n* = 52) and Bethesda VI (*n* = 51) (37), and Bethesda III/IV samples from Afirma GEC (*n* = 247).

An additional 695 blinded FNAs from the Veracyte CLIA laboratory with sufficient RNA were deidentified and examined for fusions, during which time the total rate of assay failure among all samples received was 3.85%. This blinded and consecutive cohort was chosen without bias to represent an XA intended use cohort (*n* = 634 Bethesda III/IV GSC suspicious and *n* = 61 Bethesda V/VI).

### Statistics

To evaluate the agreement when comparing the whole transcriptome RNA-seq to targeted AmpliSeq panels, positive percent agreement (PPA), negative percent agreement (NPA) and confirmation were calculated following the FDA guideline^10^. Statistical analyses were performed using R statistical software version 3.2.3^11^. All confidence intervals are 2-sided 95% CIs and were computed using the exact binomial test. The chi-square test of independence was performed to examine if there is a relationship between two categorial variables.

## Results

### A High Proportion of Variants Observed in DNA Are Expressed in RNA

To determine how many DNA variants could be detected in expressed RNA, RNA and DNA were first extracted from the same biological sample for direct comparison. Afirma GSC data was generated from the RNA, which utilizes whole-transcriptome RNA-seq data as an input to the machine learning algorithms. DNA was analyzed with a custom, targeted AmpliSeq panel that covers 761 variants that have been described in thyroid samples.

Nine hundred forty-three FNA samples were analyzed with the custom DNA AmpliSeq panel. Four hundred forty-two samples were used for parameter tuning to ensure accurate detection of variants, and after the analysis pipeline was locked, the remaining 501 samples were used to evaluate the performance of calling a variant from RNA-seq data relative to DNA-based detection. Using a DNA variant allele frequency (VAF) cutoff of 5%, 181 DNA variants were observed, and the same variants were observed in 134 RNA-seq samples (74%; Table 2). Using a 20% VAF threshold for a positive result in DNA, positive percent agreement (PPA) increases to 88%.

**Table 2:** Variant and Fusion Performance in whole transcriptome RNA-seq compared to targeted AmpliSeq panels.

**Genomic alteration**	**Samples**	**PPA**	**NPA**	**Confirmation**	**RNA-seq only**	**AmpliSeq only**	**Both detected**
DNA Variants	501	74% [67–80]	100% [100–100]	98.5% [95–100]	2	47	134
RNA Variants	102	88.9% [80–95]	100% [100–100]	94.7% [87–99]	4	9	72
Fusions	695	82% [70–91]	100% [100–100]	100% [93–100]	0	11	50

*Positive Percent Agreement (PPA) is percentage of variants or fusions that are positive by RNA-seq compared to AmpliSeq. Negative Percent Agreement (NPA) is the percentage of variants or fusions that are negative by RNA-seq compared to AmpliSeq. Confirmation is the percentage of variants or fusions that were called positive by RNA-seq that are also positive by AmpliSeq*.

### Low Levels of Variant Allele Expression May Account for Some Differences in Variant Detection Between RNA and DNA Methods

**Figure 1:**
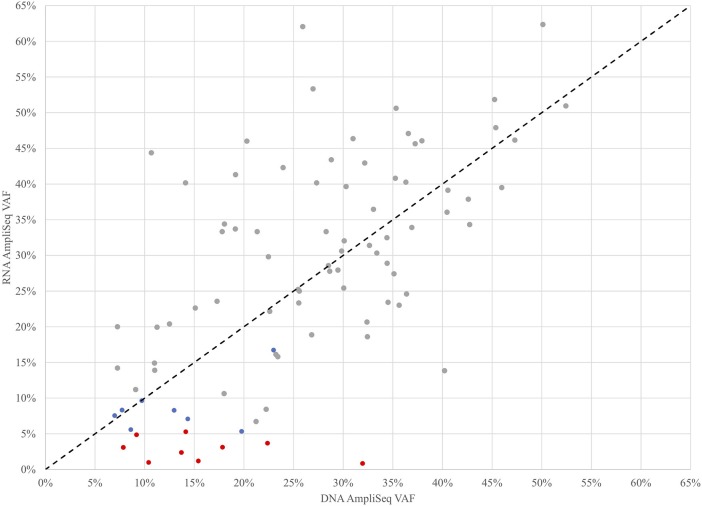
Variant Allele Frequencies (VAF) determined by targeted DNA and RNA AmpliSeq methods for 102 FNAs that were variant positive by DNA AmpliSeq. Samples with low RNA AmpliSeq coverage of the variant were excluded. Gray points were detected by all 3 methods, blue points were detected by DNA AmpliSeq and RNA AmpliSeq, but not detected by XA, red points were detected by DNA AmpliSeq but not RNA AmpliSeq or XA. The black dotted line is x=y.

To further investigate the role of transcription in the detection of expressed variants, a targeted variant panel using RNA as the template was employed rather than DNA. From the 943 FNA samples, a representative set of 102 FNAs that were variant positive by DNA AmpliSeq were tested for expressed variants with an RNA AmpliSeq variant panel. The PPA of RNA-seq whole-transcriptome variants rose from 76.5% vs. DNA in this subset to 88.9% vs. RNA (Table 2). Next, RNA Variant AmpliSeq data was examined for 17 samples that had a DNA variant identified, but no variant identified in the whole-transcriptome RNA-seq. Six of these 17 samples had dramatically different VAFs when comparing the DNA and RNA (Figure 1), with VAFs observed in DNA >10% while RNA-based VAFs were <5%. These six samples had a DNA:RNA VAF ratio ranging from 5.7 to 38.1 (including 3 samples with a DNA:RNA VAF ratio >10). In these six samples, the wild type allele is predominantly expressed and that biological difference accounts for the lack of variant detection in the whole-transcriptome RNA-seq data. In the most striking sample, we observed a VAF of 32% in the DNA and <1% in RNA.

### There Is High Agreement Between Fusion Detection by Whole-Transcriptome RNA-Seq and Targeted Fusion Seq

To determine the PPA of the fusion-calling capabilities of whole-transcriptome RNA-seq data, an in-house custom RNA fusion AmpliSeq assay was developed. A new series of 695 consecutive and blinded FNAs from the Veracyte CLIA stream that were either Afirma GSC Suspicious or Bethesda V/VI were tested for fusions with the RNA fusion AmpliSeq assay. Any sample that generated a discordant result between whole-transcriptome RNA-seq and the RNA fusion AmpliSeq assay were resolved with qPCR. A total of 61 fusions were observed in this series, and the fusions observed in greater than one FNA were: *PAX8/PPARG* (*n* = 16), *ETV6/NTRK3* (*n* = 13), *RET/PTC1* (*n* = 6), *STRN/ALK* (*n* = 6), *RET/PTC3* (*n* = 3), *AGK/BRAF* (*n* = 3), *SND1/BRAF* (*n* = 2), and *RBPMS/NTRK3* (*n* = 2) (Supplementary Table 1). This analysis revealed an 82.1% PPA between the RNA fusion AmpliSeq assay and whole-transcriptome RNA-seq data and demonstrated a 100% confirmation rate, as all 50 fusions identified by RNA-seq were also identified by the AmpliSeq assay (Table 2).

### Analytical Validation Shows the Variant and Fusion Calls Are Highly Reproducible Across Labs and Reagent Lots

To determine the reproducibility of the whole-transcriptome RNA-seq assay across laboratories and reagent lots, we examined the analytical validity of the assay. We compared the variant and fusion calls from RNA-seq data between two labs (R&D and CLIA) with the same lot of library prep reagents. Sixty-nine variant positive samples were used, and the between lab accuracy was 89.9% (Supplementary Figure 1A). For fusions, 36 positive samples were used, and the between lab accuracy was 94.4% (Supplementary Figure 1B). Next, we examined the reproducibility of the assay within one plate and across different reagent lots. Nine variant positive samples and 6 fusion positive samples were plated in triplicate across 3 plates. Each plate was run with different reagent lots and different operators. These experiments investigated intra-plate and inter-plate reproducibility. For variants, the intra-plate reproducibility was 88.9% and the inter-plate reproducibility was 86.4%. For fusions, the intra-plate reproducibility was 94.4% and the inter-plate reproducibility was 90.7%. These results passed pre-specified acceptance criteria for these studies.

### Among Bethesda III/IV Nodules, 49% of Malignancies Harbor an RNA-Seq Detected Variant or Fusion

**Figure 2:**
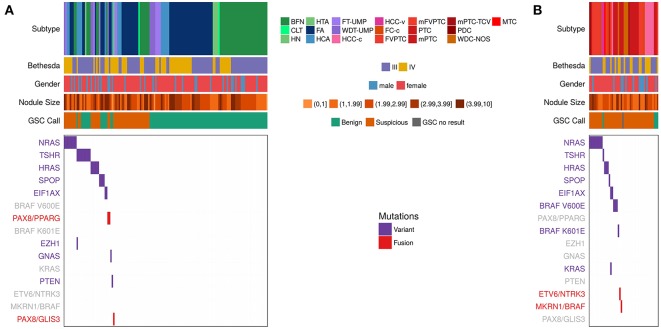
Expressed Variants and fusions observed in RNA-seq data among the n = 191 Afirma GSC clinical validation study cohort, including the 1 sample classified as GSC no result due to inadequate follicular content (25), which is a subset of the 943. Each column represents a sample, while each row is either clinical information or a variant or fusion. Samples positive for a variant have a purple hash, while samples positive for a fusion have a red hash. Negative for a variant or fusion are white. Clinical information includes subtype, Bethesda category (III and IV only), Nodule size in cm, and GSC call. See Table 1 for a list of subtype abbreviations. Variants/fusions are grayed out if no samples in a given cohort were positive for that alteration. **(A)** Histologically benign subtypes. **(B)** Histologically malignant subtypes.

To understand the relationship between variants and fusions and histopathology diagnosis, we compared the variants/fusions observed in RNA-seq data from the primary test set of 190 Bethesda III/IV samples (Figure 2 and Table 3) that were collected in a prospective, multicenter, and blinded protocol for the clinical validation of the Afirma GEC (38), and subsequently utilized to clinically validate the Afirma GSC (25). The histopathological diagnosis of these nodules was assigned by an expert panel of thyroid histopathologists who were masked to all clinical, molecular, and cytological data. In 145 histologically benign nodules, 76% had no variant or fusion observed (76% specificity). Of the 24% with a variant, *RAS* variants were the most common, followed by *TSHR*. In the 45 histologically malignant samples, 51% had no variant or fusion observed (49% sensitivity). Overall, the RNA-seq PPV and NPV were 38% and 83%, respectively, in this cohort with a 24% cancer prevalence. The 49% of samples with a positive variant or fusion mostly harbored *RAS* variants, *BRAF* variants, or fusions. Of the variants observed more than once, only *BRAF* V600E was confined to malignant nodules. Conversely, 10 of 11 *TSHR* variants and 4 of 5 *SPOP* variants occurred in histologically benign nodules. The only gene fusion observed more than once was *PAX8/PPARG* and both occurrences were in histologically benign nodules. *MKRN1/BRAF* and *ETV6/NTRK3* fusions were each identified once and were in malignant nodules (both PTC). *PAX8/GLIS3* was identified in the one hyalinizing trabecular adenoma, consistent with a recent report (39).

**Table 3:** Variants and fusions relative to their histopathologic and Afirma GSC outcomes (25). GSC Benign (B), GSC Suspicious (S).

			**XA variant and fusions (*n*)**	
**Histopathological subtype**	**Nodules, No. (%)**	**GSC B No./GSC S No**.	**GSC benign**	**GSC suspicious**
**BENIGN**				
Total	145 (100)	99/46		
BFN	49 (33.8)	38/11	EIF1AX:p.G8R(1)	HRAS:p.Q61R(2)
			TSHR:p.I486M(1)	NRAS:p.Q61K(1)
			TSHR:p.L512R(1)	NRAS:p.Q61R(1)
			TSHR:p.M453T(1)	
HN	5 (3.4)	5/0	TSHR:p.I568T(1)	NA
FA	54 (37.2)	37/17	GNAS:p.Q870H(1)	HRAS:p.Q61R(1)
			SPOP:p.P94R(3)	NRAS:p.Q61R(3)
			TSHR:p.D633Y(1)	PAX8/PPARG(1)
			TSHR:p.L629F(1)	
FT-UMP	9 (6.2)	4/5	NA	HRAS:p.Q61R(1)
				NRAS:p.Q61R(1)
				SPOP:p.P94R(1)
WDT-UMP	8 (5.5)	4/4	NA	HRAS:p.Q61R(2)
				NRAS:p.Q61R(1)
				PAX8/PPARG(1)
HCA	17 (11.7)	10/7	EIF1AX:p.G9D(1)	NRAS:p.Q61K(2)
			PTEN:p.G129R(1)	TSHR:p.M453T(1)
			TSHR:p.L629F, EZH1:p.Y642F(1)	TSHR:p.S281I(1)
			TSHR:p.S425I(1)	
CLT	2 (1.4)	1/1	NA	NA
HTA	1 (0.7)	0/1	NA	PAX8/GLIS3(1)
**MALIGNANT**				
Total	45 (100)	4/41		
PTC	15 (33.3)	2/13	NA	BRAF:p.V600E(3)
				NRAS:p.Q61R(1)
				SPOP:p.P94R(1)
				MKRN1/BRAF (1)
				ETV6/NTRK3(1)
PTC-TCV	1 (2.2)	0/1	NA	NA
FV-PTC	11 (24.4)	1/10	NA	HRAS:p.Q61R(1)
				KRAS:p.Q61R, EIF1AX:p.A113_splice(1)
				NRAS:p.Q61K(1)
				NRAS:p.Q61R(4)
HCC-c	9 (20)	1/8	TSHR:p.I568T(1)	EIF1AX:p.A113_splice(1)
				NRAS:p.Q61R(1)
FC	7 (15.6)	0/7	NA	BRAF:p.K601E(1)
				HRAS:p.G13R(1)
				NRAS:p.Q61R(1)
PDTC	1 (2.2)	0/1	NA	NRAS:p.Q61K(1)
MTC	1 (2.2)	0/1	NA	HRAS:p.Q61R(1)

We next examined the combination of Afirma GSC classifier prediction and variant/fusion results (Figure 2 and Table 3). In the 99 histologically benign nodules with GSC benign results (true negatives), 15% were variant positive and none contained a fusion. In the 46 benign nodules with GSC suspicious results (false positives), 39% harbored a variant and 6.5% contained a fusion (2 *PAX8/PPARG* and 1 *PAX8/GLIS3*). In the 41 histologically malignant nodules with GSC suspicious results (true positives), 46% had a variant, and 5% had a fusion (*MKRN1/BRAF* and *ETV6/NTRK3*). Finally, in the 4 GSC benign false negative nodules (2 PTC, 1 FVPTC, 1 HCC), only the HCC contained a variant (*TSHR*). Taken together, in the 190 thyroid nodules with definitive histology, malignant nodules were twice as likely to carry a variant or fusion relative to benign nodules (49 vs. 24%, *p* = 0.003 [χ2]). The most common genomic alteration identified among GSC suspicious nodules was a variant in a *RAS* family gene (present in 32% and conveying a PPV of 46%). Taken together, GSC suspicious nodules carried a variant or fusion in 48% compared with 16% of GSC benign nodules (*p* < 0.0001). The most common variants among GSC benign nodules were *TSHR*, and no variants or fusions known to be highly predictive of malignancy were identified among nodules with GSC benign results. In clinical use, variants and fusions are not reported among GSC benign nodules.

Among GSC suspicious nodules with a cancer prevalence of 47%, the sensitivity, specificity, and NPV of XA were 51, 54, and 56%, respectively. The overall PPV of an Afirma GSC suspicious nodule was 47%, regardless of variant/fusion status. The PPV was 50% among GSC suspicious nodules when a variant or fusions was identified, compared with 44% among GSC suspicious nodules when no variant or fusion was identified (*p* = 0.77 [χ2]).

### Variants and Fusions Potentially Amenable to Targeted Therapy Were Identified Across Multiple Bethesda Categories

**Figure 3:**
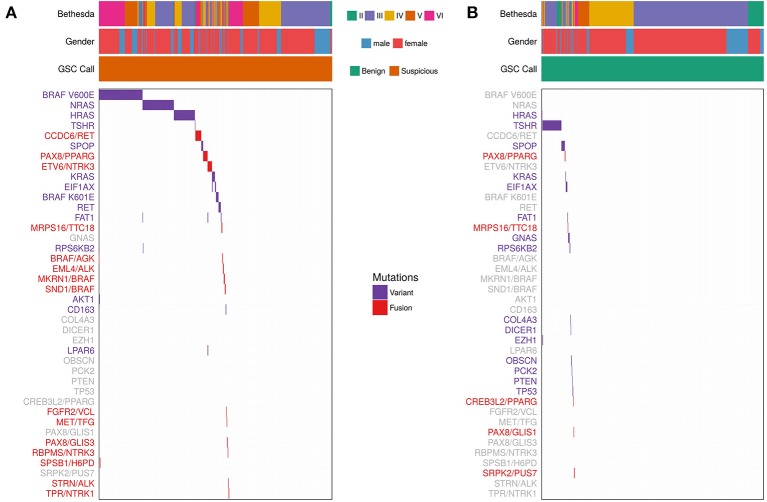
Expressed Variants and Fusions from 943 FNAs (See Materials and Methods). Each column represents one sample and each row represents variants observed in one gene or fusion pair. Bethesda category, gender, and Afirma GSC call are represented for each sample. Purple indicates that a sample is positive for a variant. Red indicates that a sample is positive for a fusion. Variants/fusions are grayed out if no samples in a given cohort were positive for that alteration. **(A)** FNAs with a GSC Suspicious call. **(B)** FNAs with a GSC Benign call.

We determined the most common variants observed in thyroid FNAs from expressed variant data (Figure 3 and Supplementary Table 2). Five genes showed variants in >1% of FNAs: *BRAF, NRAS, HRAS, TSHR*, and *SPOP*. For individual variants, *BRAF* V600E was most common, followed by *NRAS* Q61R, *HRAS* Q61R, *NRAS* Q61K, *TSHR* M453T, and *SPOP* P94R (Supplementary Table 2). *NRAS* and *HRAS* variants were primarily observed in Bethesda III/IV FNAs that were GSC Suspicious, while *BRAF* V600E was predominantly present in Bethesda V/VI FNAs. *TSHR* and *SPOP* variants were most frequently observed in GSC Benign FNAs (Figure 3), which is consistent with other observations (11, 40).

Fusions were also identified using whole-transcriptome RNA-seq. Fifty-two (5.5%) of the 943 samples harbored a fusion. The most common fusions were *CCDC6/RET* (aka *RET/PTC1*), *PAX8/PPARG*, and *ETV6/NTRK3* (Figure 3 and Supplementary Table 3). *RET/PTC1* was primarily observed in Bethesda V/VI FNAs, while *PAX8/PPARG* was observed in Bethesda III/IV FNAs. *ETV6/NTRK3* was observed across Bethesda III-VI. A subset of 31 fusion-containing samples with sufficient RNA were selected for testing by qPCR (Supplementary Table 4). 100% of the RNA-seq detected fusions were also detected (i.e., confirmed) by qPCR. *ALK* fusions were also observed, although they were rare. *EML4/ALK* was observed twice in Bethesda VI samples and *STRN/ALK* was observed once in Bethesda III.

Overall, variants or fusions potentially amenable to targeted therapy involving *AKT, ALK, BRAF, HRAS, KRAS, MET, NRAS, NTRK, PPARG, PTEN*, and *RET* were identified across multiple Bethesda categories. Similarly, 4 *RET* point mutations suggestive of MTC were identified among Bethesda III-V samples (Supplementary Table 2). All 4 were identified as positive by the Afirma GSC MTC classifier (25, 41). Further, these variants may be somatic or germline and raise the possibility of multiple endocrine neoplasia type 2.

### 1% of Bethesda III/IV Nodules Harbor a TERT Promoter Variant, All Were in Combination With a RAS Variant and Were GSC Suspicious, and Most Were Histologically Benign

The DNA AmpliSeq panel included the *TERT* promoter. *TERT* promoter variants were observed in 15 samples of the 943 examined, across Bethesda categories (Table 4), and all were called Afirma GSC suspicious: 1 Bethesda II (1.89%), 0 Bethesda III (0%), 7 Bethesda IV (3.48%), 2 Bethesda V (1.90%), and 6 Bethesda VI (5.61%). The Bethesda II sample was *TERT* C228T plus *NRAS* Q61K. All 7 Bethesda IV FNAs were *TERT* C228T plus *RAS* positive. Seven of eight Bethesda V and VI FNAs were *TERT* C228T plus *BRAF* V600E, with the remaining Bethesda VI sample *TERT* C228T positive in isolation. Fourteen of fifteen *TERT* C228T positive FNAs had paired histopathology (Table 4). The Bethesda II sample was an FVPTC. In Bethesda IV, 5 FNAs were *TERT* C228T plus *NRAS* positive (4 Q61R and 1 Q61K) and 4/5 were histologically benign. Additionally, there were 2 FNAs that were positive for *TERT* C228T in combination with *KRAS* Q61R or *HRAS* Q61R, which were histologically malignant and benign, respectively. Finally, five Bethesda V and VI FNAs were *TERT* C228T in combination with *BRAF* V600E, and all 5 were histologically malignant. Three were PTC and two were PTC-TCV. The Bethesda VI sample with *TERT* C228T in isolation was an FC-v.

**Table 4:** *TERT* Promoter variants observed in this study.

**Bethesda**	**Histopathology**	**SubType**	**Afirma GSC**	**Variant**	**TERT Promoter Variant**
Bethesda II	M	FVPTC	Suspicious	*NRAS*:p.Q61K	C228T
Bethesda IV	B	HCA	Suspicious	*HRAS*:p.Q61R	C228T
	B	HCA	Suspicious	*NRAS*:p.Q61K	C228T
	B	NHP	Suspicious	*NRAS*:p.Q61R	C228T
	B	FA	Suspicious	*NRAS*:p.Q61R	C228T
	B	WDT-UMP	Suspicious	*NRAS*:p.Q61R	C228T
	M	PTC	Suspicious	*KRAS*:p.Q61R	C228T
	M	FC-v	Suspicious	*NRAS*:p.Q61R	C228T
Bethesda V	M	PTC	Suspicious	*BRAF*:p.V600E	C228T
	M	PTC	Suspicious	*BRAF*:p.V600E	C228T
Bethesda VI	M	PTC	Suspicious	*BRAF*:p.V600E	C228T
	M	PTC-TCV	Suspicious	*BRAF*:p.V600E	C228T
	M	PTC-TCV	Suspicious	*BRAF*:p.V600E	C228T
	Unknown	Unknown	Suspicious	*BRAF*:p.V600E	C228T
	M	FC-v	Suspicious	None	C228T

*Histopathology is shown as Malignant (M) or Benign (B). See Table 1 for a list of subtype abbreviations. Unknown subtype has no histopathology information available*.

## Discussion

This study demonstrates the analytical and clinical validation in thyroid nodule evaluation of the Afirma Xpression Atlas (XA), which detects gene variants and fusions from a curated panel of 511 genes via dedicated FNA samples using whole-transcriptome RNA-sequencing. More than 96% of consecutive real-world samples received has sufficient quantity and quality to receive an XA result. The data show that agreement is high between fusion detection by XA and an alternative fusion detection method, and that a high proportion of variants observed in DNA are also detected in the expressed RNA. As expected, agreement is even higher when comparing variant detection between two different RNA-based methods. Intriguingly, we found the wild type allele to be preferentially expressed compared with the variant allele in some samples (Figure 1), explaining some of the differences observed. Analytical studies show high reproducibility within plates and across reagent lots. Among Bethesda III/IV cytologically indeterminate nodules that were malignant, about half harbored a variant or fusion that was detected by XA. Detection of variants and fusions progressively increased along the Bethesda II to VI spectrum, and genomic findings potentially amenable to targeted therapeutics were identified across the Bethesda spectrum.

Afirma XA is not a cancer rule-out test. Among Bethesda III/IV nodules deemed suspicious by Afirma GSC and among Bethesda V/VI nodules, the impact of Afirma XA on nodule management extends beyond informing the risk of cancer when XA is negative or when XA is positive for a specific genomic alteration. Recent studies have begun to associate selected variant and fusions with *BRAF* V600E-like vs. *RAS*-like (or non-BRAF-non-RAS) pathway signaling, iodine metabolism, neoplasm histology, risk of lymph node metastasis, risk of recurrence, and risk of mortality (3, 9). How much these prognostic associations will remain significant independent predictors when traditional predictors are considered is presently unknown. For instance, *TERT* promoter mutations predict disease-free survival and disease-specific survival, but this effect is diminished or eliminated when the variant occurs in the absence of a *RAS* or *BRAF* variant, or among low and intermediate ATA risk patients and stage I-II TNM patients (42). Thus, for thyroid nodules seemingly confined to the thyroid gland and harboring Bethesda III-VI cytology, future studies may investigate how the preoperative identification of a presumed driver mutation may inform the pre-operative evaluation and surgical plan. For example, prospective randomized trials could investigate the roles of active surveillance or hemithyroidectomy for variants and fusions associated with a ~50:50 chance of cancer, and/or those associated with less aggressive carcinomas. This may include nodules where a variant or fusion is not detected as some data suggests that such cancers may have less aggressive features when considering extrathyroidal extension, lymph node metastases, risk of recurrence, and risk of mortality (3, 9). Similarly, prospective randomized trials could investigate the role of total thyroidectomy vs. hemithyroidectomy (or whether or not adjuvant radioactive iodine ablation or TSH suppression would be used) for cancers with variants thought to predict more aggressive tumor behavior that are nevertheless clinically confined to the thyroid gland.

For patients presenting with locally advanced thyroid cancer, genomic findings on XA may suggest the possibility of neo-adjuvant therapy that may improve the outcome of a subsequent surgical resection (16, 43, 44).

For patients with thyroid carcinoma in the neck or in distant sites that is refractory to radioactive iodine and may warrant systemic therapy, use of XA from FNAs of known thyroid cancer deposits may inform treatment selection, although confirmation testing by an approved companion diagnostic test may still be required for patients to access certain pharmaceuticals. Additionally, repeat testing from a site of disease progression during active treatment may provide additional genomic insights to potentially guide therapy (26).

Limitations of measuring variants in expressed RNA include that some variants and fusions identified by an alternative method were not identified by XA. The reason for these differences is not known, nor is it known which test should ideally be considered “correct.” While imperfect test sensitivity is one possibility, it is also possible that some DNA variants may not be expressed due to gene silencing, or very low expression levels. Such phenomenon may explain some discrepancies among *BRAF* V600E variants detected by qPCR that are negative by immunohistochemistry (45). By employing a third variant detection methodology that used targeted sequencing of RNA templates, we have shown that some samples do have very low expression of the gene variants identified in DNA. The biological significance of such variants is unknown. The efficacy of targeted treatment aimed at non-expressed or poorly expressed genomic alterations may be diminished. Conversely, the vast majority of genomic abnormalities identified by XA were confirmed by the alternative method (Table 2). An additional limitation of RNA sequencing is that variants in non-coding regions, such as *TERT* promotor variants, are not detected by this method. However, our data demonstrate that these variants are uncommon among cytologically indeterminate nodules (<1%) and in the vast majority of cases, found in tandem with a *RAS* variant. Moreover, we show that *TERT* promoter variants in combination with *RAS* variants can occur in benign lesions in Bethesda IV FNAs. While current opinion is that nodules with a *RAS* variant (with or without *TERT* promoter variant) should be surgically removed given their potential malignant or pre-malignant status, it is unclear if cancers harboring a *TERT* promoter variant plus a *RAS* or *BRAF* variant should be treated differently based on this genomic information independent from traditional prognostic factors for risks of recurrence and death, especially among lower-risk patients (42). A DNA based detection method, or development of an RNA expression-based classifier, could be added to XA in the future should reporting of such non-expressed variants be desired.

In clinical practice, XA testing is offered for Bethesda III/IV Afirma GSC suspicious nodules, FNA Bethesda V/VI nodules, and for known thyroid cancer metastases. The Afirma Xpression Atlas became commercially available to most of the United States in 2018. Samples for Afirma GSC and/or XA are collected with two dedicated FNA needle passes that must be expressed into the provided FNAprotect tube, properly stored locally to avoid exposure to heat, and are shipped in the provided container with frozen foam bricks to ensure receipt of high-quality RNA material. These validated collection and shipping procedures are identical to those used to formerly collect and ship Afirma GEC samples (46). Detailed sample collection, packing, and shipping instructions are available on-line^12^.

In summary, we have demonstrated clinical and analytical validation of the Afirma XA, which reports variants and fusions from a panel of 511 genes that have been associated with thyroid cancer. This added clinical information is intended to supplement clinical decision-making among patients with Bethesda III-VI nodules. Clinicians are to be reminded that most patients with thyroid cancer have an excellent prognosis, and the greatest impact of this added genomic information may be to facilitate treatment that is less aggressive, rather than more aggressive (47). The information obtained from variants and fusions assessment may offer new precision medicine insights from diagnostic FNA samples and the opportunity to advance individualized patient care.

## Data Availability

Restrictions apply to the datasets: The datasets for this manuscript are not publicly available because the dataset and the research methodologies are proprietary. Requests to access the datasets should be directed to GK, Ph.D.; Giulia@Veracyte.com.

## Ethics Statement

This research was approved by the Copernicus Group Independent Review Board (Cary, North Carolina). A waiver of written informed consent was granted regarding de-identified biological materials from the CLIA laboratory. IRB approval and written Informed consent in accordance with the Declaration of Helsinki was provided by all patients whose samples were previously used for training and validation of the Afirma GSC as previously described (25, 38).

## Author Contributions

GK, JB, JH, PW, SK, and YH conceived and designed the study. JB, LW, PW, RK, SK, TA, and YH analyzed and interpreted the data. EC, GK, JB, LW, MC, MS, RK, SW, and TA drafted and critically revised the work for important intellectual content. SK and YH performed the bioinformatics and statistical analyses. All authors contributed to manuscript revision, read and approved the submitted version.

### Conflict of Interest Statement

GK, JB, JH, PW, RK, SK and YH are Veracyte Inc. employees and equity owners. The remaining authors declare that the research was conducted in the absence of any commercial or financial relationships that could be construed as a potential conflict of interest.

## Footnotes

^1^https://clinicaltrials.gov/ct2/show/NCT02465060

^2^https://clinicaltrials.gov/ct2/show/NCT03065387

^3^https://clinicaltrials.gov/ct2/show/NCT02383927

^4^https://clinicaltrials.gov/ct2/show/NCT03244956

^5^https://clinicaltrials.gov/ct2/show/NCT03181100

^6^https://clinicaltrials.gov/ct2/show/NCT00019331

^7^https://clinicaltrials.gov/ct2/show/NCT03600883

^8^https://clinicaltrials.gov/ct2/show/NCT03785249

^9^https://clinicaltrials.gov/ct2/show/NCT01655719

^10^https://www.fda.gov/RegulatoryInformation/Guidances/ucm071148.htm section 7.2

^11^https://www.r-project.org

^12^www.afirma.com/physicians/practice-resources
