# Supplementary material for: Analytical and Clinical Validation of Expressed Variants and Fusions From the Whole Transcriptome of Thyroid FNA Samples
Source: Front Endocrinol (Lausanne). 2019 Sep 11;10:612. doi: 10.3389/fendo.2019.00612 (PMC6749016; doi:10.3389/fendo.2019.00612)
Supplement: Supplementary file 1 [file Data_Sheet_1.PDF]

**A.**

| | | R&D Lab | | |
|---|---|---|---|---|
| | | **Not-Detected** | **Positive** | **Sum** |
| **CLIA Lab** | **Not-Detected** | 20 | 7 | **27** |
| | **Positive** | 8 | 62 | **70** |
| | **Sum** | **28** | **69** | **97** |

**B.**

| | | R&D Lab | | |
|---|---|---|---|---|
| | | **Not-Detected** | **Positive** | **Sum** |
| **CLIA Lab** | **Not-Detected** | 4 | 2 | **6** |
| | **Positive** | 2 | 34 | **36** |
| | **Sum** | **6** | **36** | **42** |

**Supplementary Figure 1**. Results from the lab-to-lab accuracy study. The 2x2 tables show variant or fusion positive samples for each lab, and variant and fusion not-detected samples in each lab. Both labs used the same reagents, but the laboratory and personnel varied. **A.** Variant-positive samples. **B.** Fusion-positive samples.

**Supplementary Table 1**. Fusions observed in 695 consecutive FNAs

| **Fusion ID** | **Count** |
|---|---|
| *PAX8/PPARG* | 16 |
| *ETV6/NTRK3* | 13 |
| *STRN/ALK* | 6 |
| *CCDC6/RET (RET/PTC1)* | 6 |
| *AGK/BRAF* | 3 |
| *NCOA4/RET (RET/PTC3)* | 3 |
| *SND1/BRAF* | 2 |
| *RBPMS/NTRK3* | 2 |
| *EML4/AML* | 1 |
| *MACF1/BRAF* | 1 |
| *MKRN1/BRAF* | 1 |
| *POR/BRAF* | 1 |
| *HTATSF1/BRS3* | 1 |
| *CREB3L2/PPARG* | 1 |
| *DNAJC6/PDE1C* | 1 |
| *IGF2BP3/THADA* | 1 |
| *IQGAP1/ZNF774* | 1 |
| *TFG/MET* | 1 |
| Total | 61 |

**Supplementary Table 2**. Variants observed in RNA-seq data among 943 samples. Each row is a variant observed in RNA-seq data and each column represents that Bethesda category.

| Row Labels | Bethesda II | Bethesda III | Bethesda IV | Bethesda V | Bethesda VI | Grand Total |
|---|---|---|---|---|---|---|
| None | 38 | 359 | 148 | 67 | 47 | 659 |
| *BRAF:p.V600E* | 2 | 6 | 2 | 26 | 52 | 88 |
| *NRAS:p.Q61R* | | 25 | 13 | 3 | 3 | 44 |
| *HRAS:p.Q61R* | 1 | 18 | 12 | 1 | 1 | 33 |
| *NRAS:p.Q61K* | 1 | 12 | 4 | 1 | | 18 |
| *TSHR:p.M453T* | 3 | 11 | | | | 14 |
| *SPOP:p.P94R* | | 7 | 2 | 2 | | 11 |
| *HRAS:p.Q61K* | 1 | 6 | 2 | | | 9 |
| *TSHR:p.I486F* | 1 | 5 | | 2 | | 8 |
| *BRAF:p.K601E* | | 3 | 1 | 1 | | 5 |
| *TSHR:p.T632I* | | 3 | | | | 3 |
| *HRAS:p.G13R* | | 3 | | | | 3 |
| *TSHR:p.D633Y* | 2 | 1 | | | | 3 |
| *GNAS:p.Q870H* | | 2 | 1 | | | 3 |
| *TSHR:p.I568T* | 1 | 1 | 1 | | | 3 |
| *TSHR:p.I568F* | | 1 | 1 | | | 2 |
| *KRAS:p.Q61K* | | | | | 2 | 2 |
| *FAT1:p.V912I* | | | 2 | | | 2 |
| *KRAS:p.G12V* | | 1 | 1 | | | 2 |
| *EIF1AX:p.G9D* | | 1 | 1 | | | 2 |
| *TP53:p.P278L* | | | 1 | | | 1 |
| *RET:p.C634W* | | | | 1 | | 1 |
| *RET:p.A883F* | | | | 1 | | 1 |
| *BRAF:p.V600E,AKT1:p.E17K* | | | | | 1 | 1 |
| *RPS6KB2:p.R364W* | | 1 | | | | 1 |
| *EIF1AX:p.A113_splice* | | 1 | | | | 1 |
| *TSHR:p.D633H* | | 1 | | | | 1 |
| *FAT1:p.V912I, LPAR6:p.E318K* | | | | | 1 | 1 |
| *RET:p.C634R* | | 1 | | | | 1 |
| *TSHR:p.S505N* | 1 | | | | | 1 |
| *RET:p.M918T* | | | 1 | | | 1 |
| *KRAS:p.G12D* | | 1 | | | | 1 |
| *EIF1AX:p.G8R* | | | 1 | | | 1 |
| *COL4A3:p.T255M* | 1 | | | | | 1 |
| *TSHR:p.D633E* | | 1 | | | | 1 |
| *TSHR:p.I486M* | | 1 | | | | 1 |

| | | | | | | |
|---|---|---|---|---|---|---|
| *KRAS:p.Q61R, EIF1AX:p.A113_splice* | | | 1 | | | 1 |
| *KRAS:p.G12R* | | | 1 | | | 1 |
| *TSHR:p.L512R* | 1 | | | | | 1 |
| *DICER1:p.D1810H* | | 1 | | | | 1 |
| *EIF1AX:p.G9R* | | | 1 | | | 1 |
| *CD163:p.S505L* | | 1 | | | | 1 |
| *TSHR:p.L629F, EZH1:p.Y642F* | | | 1 | | | 1 |
| *OBSCN:p.E1373K* | | 1 | | | | 1 |
| *TSHR:p.S425I* | | 1 | | | | 1 |
| *PCK2:p.I268V* | | | 1 | | | 1 |
| *PTEN:p.G129R* | | | 1 | | | 1 |
| *NRAS:p.Q61R, RPS6KB2:p.R364W* | | 1 | | | | 1 |
| *NRAS:p.Q61R, FAT1:p.V912I* | | | 1 | | | 1 |
| **Grand Total** | **53** | **477** | **201** | **105** | **107** | **943** |

**Supplementary Table 3**. Fusions observed in 943 samples. Each row is a gene fusion observed in RNA-seq data and each column represents that Bethesda category.

| **Row Labels** | **Bethesda II** | **Bethesda III** | **Bethesda IV** | **Bethesda V** | **Bethesda VI** | **Grand Total** |
|---|---|---|---|---|---|---|
| *CCDC6/RET (RET/PTC1)* | | | | 3 | 9 | 12 |
| *PAX8/PPARG* | | 5 | 5 | | | 10 |
| *ETV6/NTRK3* | | 5 | 2 | 1 | 1 | 9 |
| *MRPS16/TTC18* | 1 | 2 | 1 | | | 4 |
| *EML4/ALK* | | | | | 2 | 2 |
| *MKRN1/BRAF* | | 1 | 1 | | | 2 |
| *SND1/BRAF* | | | | 2 | | 2 |
| *AGK/BRAF* | | | | 1 | 1 | 2 |
| *STRN/ALK* | | 1 | | | | 1 |
| *TPR/NTRK1* | | | | 1 | | 1 |
| *SRPK2/PUS7* | | | 1 | | | 1 |
| *CREB3L2/PPARG* | | 1 | | | | 1 |
| *RBPMS/NTRK3* | | 1 | | | | 1 |
| *SPSB1/H6PD* | | | | | 1 | 1 |
| *TFG/MET* | | | | 1 | | 1 |
| *PAX8/GLIS1* | | | | 1 | | 1 |
| *FGFR2/VCL* | | | 1 | | | 1 |
| **Grand Total** | **1** | **16** | **11** | **10** | **14** | **52** |

**Supplementary Table 4.** Fusions confirmed by qPCR.

| **Fusion** | **Number confirmed** |
|---|---|
| *CCDC6/RET (RET/PTC1)* | 7 |
| *ETV6/NTRK3* | 6 |
| *PAX8/PPARG* | 6 |
| *MRPS16/TTC18* | 3 |
| *EML4/ALK* | 1 |
| *AGK/BRAF* | 1 |
| *TFG/MET* | 1 |
| *RBPMS/NTRK3* | 1 |
| *MKRN1/BRAF* | 1 |
| *CREB3L2/PPARG* | 1 |
| *SRPK2/PUS7* | 1 |
| *FGFR2/VCL* | 1 |
| *STRN/ALK* | 1 |
